# Fomites and the environment did not have an important role in COVID-19 transmission in a Brazilian mid-sized city

**DOI:** 10.1038/s41598-021-95479-5

**Published:** 2021-08-05

**Authors:** Ana Luíza Silva Rocha, Josilene Ramos Pinheiro, Thamilin Costa Nakamura, José Domingos Santos da Silva, Beatriz Gonçalves Silva Rocha, Raphael Contelli Klein, Alexander Birbrair, Jaime Henrique Amorim

**Affiliations:** 1 Laboratório de Agentes Infecciosos e Vetores (LAIVE), Centro das Ciências Biológicas e da Saúde, Universidade Federal do Oeste da Bahia, Rua da Prainha, 1326, Morada Nobre, Barreiras, BA CEP 47810-047 Brazil; 2 Programa de Pós-Graduação em Biologia e Biotecnologia de Microrganismos, Universidade Estadual de Santa Cruz, Ilhéus, BA Brazil; 3 Programa de Pós-Graduação em Química Pura e Aplicada, Centro das Ciências Exatas e Tecnológicas, Universidade Federal do Oeste da Bahia, Barreiras, BA Brazil; 4 Departamento de Patologia, Instituto de Ciências Biológicas, Universidade Federal de Minas Gerais, Belo Horizonte, MG Brazil

**Keywords:** Bacteria, Virology

## Abstract

It is not clear if COVID-19 can be indirectly transmitted. It is not possible to conclude the role of the environment in transmission of SARS-CoV-2 without studying areas in which people transit in great numbers. In this work we aimed to better understand the role of environment in the spread of COVID-19. We investigated the presence of SARS-CoV-2 in fomites as well as in the air and in the sewage using RT-qPCR. We studied both, a reference market area and a COVID-19 reference hospital at Barreiras city, Brazil. We collected and analyzed a total of 418 samples from mask fronts, cell phones, paper money, card machines, sewage, air and bedding during the ascendant phase of the epidemiological curve of COVID-19 in Barreiras. As a result, we detected the human RNAse P gene in most of samples, which indicates the presence of human cells or their fragments in specimens. However, we did not detect any trace of SARS-CoV-2 in all samples analyzed. We conclude that, so far, the environment and inanimate materials did not have an important role in COVID-19 transmission in Barreiras city. Therefore, similar results can probably be found in other cities, mainly those with COVID-19 epidemiological scenarios similar to that of Barreiras city. Our study is a small piece indicating the possibility that fomites and the environment do not have an important role in COVID-19 transmission. However, further studies are necessary to better understand the world scenario.

## Introduction

COVID-19 (coronavirus disease 2019) is caused by the new coronavirus SARS-CoV-2 (*Severe acute respiratory syndrome coronavirus 2*) identified in China, in late 2019^1–3^. The disease is highly efficiently transmitted and was spread to all continents, becoming a pandemic. Current evidence suggests that SARS-CoV-2 has a natural animal origin, most likely from bats, as these animals are ecological reservoirs of these viruses^4,5^. However, it is more likely that the transmission of SARS-CoV-2 to humans happened through an intermediate host, which so far has not been identified. The presence of an intermediate host in this context would be explained by the low probability of direct transmission between bats and humans, since there is limited contact between them. As for the initial cases of COVID-19, the first patients were notified in late December 2019 and early 2020^4^. They were directly related to the Wholesale Seafood Market of Huanan in the city of Wuhan, China. Environmental samples collected from the market in December 2019 tested positive for SARS-CoV-2 and, therefore, the market was interpreted as the source of the first outbreak of COVID-19. However, subsequent investigations led to the conclusion that the first cases of humans infected with the new coronavirus showed symptoms in early December and that these people were likely to have been infected as early as November. Furthermore, these cases had no direct link to the Wuhan Wholesale Seafood Market^4^. Even from this most recent information, the question remains about the period when the virus was introduced into the human population and how it actually happened.

According to the World Health Organization (WHO), more than 186,000,000 cases with more than 4,000,000 deaths were confirmed worldwide as of July 13, 2021^6^. To date, there is no specific antiviral drug capable of efficiently controlling SARS-CoV-2 approved for use in humans, although therapies based on anti-coagulants and convalescent plasma have been shown to be promising^7–9^. Fortunately, there are several vaccine formulations approved for use in humans^10^. However, vaccination of the majority of world populations is not being achieved, which prevents the containment of the pandemic. In addition, the raising of genetic variants of SARS-CoV-2 with increased transmission capacity and immune escape potential is a relevant concern^11^, due to the possibility of both, increasing in transmission speed of COVID-19 and compromising of vaccines efficacy.

The cost of dealing with the COVID-19 is huge. Hospitalization, testing, tracing, mitigating strategies, masks and cleaning of fomites demand a new routine of increased costs for governments and businessmen and also impact the domestic budgets. The increased use of disinfectants in inanimate materials is now a new normal scenario, due to the concern regarding indirect transmission of SARS-CoV-2. By the end of 2020, a total of US$4.5 billion in disinfectants was sold, an increase of more than 30% over 2019^12^. However, it is not clear if COVID-19 can be indirectly transmitted through fomites. This is an important issue also regarding the story of animal source origins.

The presence of SARS-CoV-2 in the air and inanimate surfaces of intensive care units was reported in the middle of 2020 and in the beginning of 2021^13–15^. However, the viral load was not informed. It is not possible to understand the role of inanimate surfaces and the environment in transmission of SARS-CoV-2 without sampling in areas in which people transit in great numbers, such as market areas. Are we exaggerating the risk of transmission of COVID-19 by fomites? What is the risk of SARS-CoV-2 transmission by fomites in real-life conditions? In this work we aimed to answer these questions and contribute to the understanding regarding the role of environment in the transmission of COVID-19 using Barreiras city, Brazil, as a model.

## Materials and methods

### Ethics statement

All methods were carried out in accordance with relevant guidelines and regulations. Ethics approval was obtained from institutional review board (ethics committee) (CAAE 40779420.6.0000.8060) of the Universidade Federal do Oeste da Bahia. The need of informed consents was waived by the ethics committee due to risks involved in the pandemic situation. Epidemiological data was retrieved from the epidemiological bulletins on the website of the city's municipal health department and Instituto Brasileiro de Geografia e Estatística (IBGE)^16^. We did not have access to personal data.

### Aim and specific aims

In this study, we aimed to understand the role of the environment in the transmission of COVID-19. Specific aims were: (i) to investigate the presence of SARS-CoV-2 in inanimate objects using RT-qPCR and (ii) to investigate the presence of SARS-CoV-2 in the air and in the sewage using RT-qPCR.

### Study area

The investigation was carried out in Barreiras city, which is located in the western region of the state of Bahia, Brazil, from June 2020 to May 2021. Barreiras is the 10th largest city in the state and the largest in the west of Bahia, with more than 150,000 inhabitants. Due to its economic potential and health structure, the city receives people from all over the western region and now, in the pandemic period, it receives a relevant number of COVID-19 patients, as Barreiras has the largest number of beds in Intensive Care Units (ICU)^17^ in the western region of Bahia.

### Study design

In order to verify the presence of SARS-CoV-2 in the environment, we studied the main market area of Barreiras which includes stores, supermarkets, restaurants, snack bars, bars and a variety of commercial points. We also studied the Eurico Dutra Hospital, a city reference health unit for COVID-19. We collected samples of mask fronts, cell phones, paper money, card machines, sewage, air and bedding. Samples were kept refrigerated at 4–8 °C and analyzed up to 6 h after being collected. The study was conducted during the ascendant phase of the epidemiological curve of COVID-19 in Barreiras city. Viral detection using the RT-qPCR method was performed at the Laboratório de Agentes Infecciosos e Vetores, Universidade Federal do Oeste da Bahia, in Barreiras, Brazil. To determine the sample size we considered a margin of error of 5%. In addition, we considered the total population of Barreiras city as potential donors of samples, as previously described^18,19^. According to IBGE, the population of Barreiras city is of 156,975 inhabitants. Thus, we calculated a minimum of 383 samples in order to represent the city.

### Environmental samples

Sampling was carried out from June 1, 2020 to May 13, 2021. Four of these samplings were carried out at the main market area of Barreiras (an open public place with intense circulation of people). On June 1, 2020 (maximum temperature of 31.3 °C; minimum humidity of 49%) we collected five samples of sewage, 27 samples from cell phones and 30 samples from paper money. On June 12, 2020 (maximum temperature of 32.3 °C; minimum humidity of 33%) we collected nine samples of air, 36 samples from mask fronts, 30 samples from paper money, 10 samples from card machines and 12 samples from cell phones. On June 26, 2020 (maximum temperature of 31.6 °C; minimum humidity of 40%), at the Eurico Dutra Hospital, we collected 12 samples of air from wards that admit patients with COVID-19, as well as 27 samples of masks (including health professionals and COVID-19 patients) and 12 samples from bed linen of patients admitted with COVID-19 confirmed by RT-qPCR. We also collected samples from 12 cell phones owned by patients and health workers. On August 3, 2020 (maximum temperature of 30.3 °C; minimum humidity of 30%), again at the market area, we collected 12 samples from mask fronts, 12 samples from card machines, 30 samples from paper money and 18 samples from cell phones. And on May 13, 2021, (maximum temperature of 28.1 °C; minimum humidity of 35%), again at the market area, we collected 50 samples from card machines, 50 samples from paper money and 50 samples from cell phones. In total, we collected 418 samples from the environment during the ascendant phase of the epidemiological curve of COVID-19 in Barreiras city.

For the collection of fomites, the following materials were used: 15 mL tubes with 2 mL of sterile saline solution [NaCl 0.9% (v/v)] and sterile swabs. Swabs were rubbed on surfaces of the materials. After collection, they were put in identified tubes. Sterile Pasteur pipettes were used to collect 2 mL of sewage samples in each of five different points of the market area. Air samples were collected by retention of particles using a high volume sampler (Energética, Brazil) with polytetrafluoroethylene (PTFE) filter and a regenerated cellulose filter, each 47 mm in diameter and 0.22 μm pores. The instrument was left on for 1 h per collection and membranes were removed and placed in sterile 15 mL tubes containing 2 mL of sterile saline. Tubes containing membranes were vortexed in order to suspend air particles in sterile saline. After each collection, the samples were stored at 4–8 °C and sent for viral RNA extraction within 6 h.

### Epidemiological data

Epidemiological data of the COVID-19 pandemic scenario in Barreiras were taken from the epidemiological bulletins on the website of the city's municipal health department and from IBGE, as described above^16,20^. The numbers of total cases, active cases and deaths were used in the study and collected from the date of the first case notified in Barreiras (March 21, 2020) and completed at the time of the study closure (May 13, 2020). The epidemiological bulletins came with the following information: notified cases, positive cases, discarded cases, deaths and awaiting results. We calculated the prevalence of COVID-19 cases and deaths due to COVID-19 for each of the days in which we sampled.

### RNA extraction and RT-qPCR

The nucleic acid extraction from environmental samples was performed using the PureLink^®^ Viral RNA/DNA Mini Kit (Invitrogen), following the manufacturer’s protocol.

RT-qPCR assays were carried out using 2.5 µL of purified RNA, 4.5 µL of ultrapure H_2_O, 2.5 µL of TaqMan™ Fast Virus 1-Step Master Mix (Applied Biosystems) and 0.75 µL of primers and probes (Integrated DNA Technologies—IDT primers and probes for N1, N2 or RP assays, in CDC’s recommended working concentrations), in a final volume of 10 μL reaction. Thermocycling was carried out in a QuantStudio 5 instrument (Applied Biosystems) with a hold stage composed of a first step of 5 min at 50 °C, followed by a second step of 20 s at 95 °C. The PCR stage was composed of a first step of 15 s at 95 °C followed by a second step of 1 min at 55 °C, repeated 45 times. Cycle thresholds (CT) values ≤ 34.1 were interpreted as positive, between 34.1 and 35 as inconclusive and from 35.1 to 45 as negative for SARS-CoV-2, according to our internal standard curve, cut off and decision matrix constructed based on assays carried out with a control plasmid harboring the N gene (2019-nCoV_N_Positive Control—IDT). CT values of RT-qPCR and our standard curve were used as indicators of the copy number of SARS-CoV-2 genome copies (GC) in specimens with lower cycle threshold values corresponding to higher viral copy numbers, as previously described^21^. RT-qPCR assays used here had a limit of detection of 10 genome copies, regarding SARS-CoV-2. It corresponds to a CT value of 37.

### Testing reliability of sampling method

The virus used in this experiment is a SARS-CoV-2 and was stored in the Laboratório de Agentes Infecciosos e Vetores of the Universidade Federal do Oeste da Bahia as part of the routine molecular diagnosis of COVID-19 based on RT-qPCR. We tested two materials: glass and fabric; in two different conditions: outdoors and indoors. Temperature and humidity were measured in all conditions during the experiment and are presented in Table 1. Sterile materials (glass or fabric) were contaminated in different areas of their surfaces with an equivalent to 500,000 genome copies in 50 µL of SARS-CoV-2 suspension in each area, in triplicate. Three independent experiments were carried out. The contamination was carried out into a biological safety cabinet class II-B2 and left to air dry for 20 min. The materials were incubated into the biological safety cabinet (indoors) or at a sealed box exposed to environmental conditions (outdoors). Samples were collected with a sterile swab, as described above and submitted to RNA extraction and RT-qPCR. Samples were collected immediately after contamination and air dry of materials (point 0) and on times 1.5 h, 3 h, 6 h and 12 h.

**Table 1:** Temperature and humidity in conditions of the experiment carried out to evaluate the sampling method.

Time (h)	External environment		Internal environment	
	Temperature (°C)	Humidity (%)	Temperature (°C)	Humidity (%)
0	24.3	50	24.3	50
1.5	32.5	47	21	64
3	34.4	43	21.4	65
6	43.8	24	20.8	74
12	22.9	81	21.4	70

### Statistical analysis

We performed statistical analysis using PRISM version 5.1. We compared CT values using Analysis of Variance test (ANOVA) with Bonferroni’s multiple comparison test. A p-value ≤ 0.05 was considered as statistically significant.

## Results

### Epidemiological surveillance

As shown in Table 2, we carried out our first sampling in the beginning of the ascendance of COVID-19 curve in Barreiras city, on June 1, 2020, when COVID-19 prevalence was of 0.471 cases per 1000 inhabitants. The last sampling was carried out with a prevalence of 88.695 COVID-19 cases and 1.312 deaths per 1000 inhabitants. Collectively, these results indicate that our sampling was carried out during the active circulation of SARS-CoV-2 in Barreiras city.

**Table 2:** Prevalence of COVID-19 confirmed cases and deaths due to COVID-19 in days of sampling in Barreiras city. The prevalence is expressed as number of cases per 1000 inhabitants.

	01/06/2020	12/06/2020	26/06/2020	03/08/2020	13/05/2021
Prevalence of COVID-19 cases	0.471	0.949	2.057	10.460	88.695
Prevalence of deaths due to COVID-19	0	0	0.031	0.159	1.312

### Lack of detection of SARS-CoV-2 in the environment during ascendant curve of COVID-19 in Barreiras city

As shown in Table 3, the human RNAse P gene was detected in almost all samples, indicating the presence of human cells or their fragments in most of specimens. However, SARS-CoV-2 RNA (any trace) was not detected in any kind of sample collected in both, the market area and the COVID-19 reference hospital. This is a striking result that indicates that the virus was not present in the sampled environmental specimens and inanimate objects during the ascendant curve of COVID-19 cases in Barreiras city.

**Table 3:** Mean and standard deviation of Cycle thresholds (CT) of the RNAse P gene detected by RT-qPCR in samples collected.

	Samples						
	Mask	Cell phone	Paper money	Sewage	Air	Bedding	Card machine
Mean	36,592	35,140	34,138	37,004	0	0	32,753
STD*	3746	2488	2288	3430	0	0	7440

^*^STD standard deviation.

### Proof of reliability of sampling method

**Figure 1:**
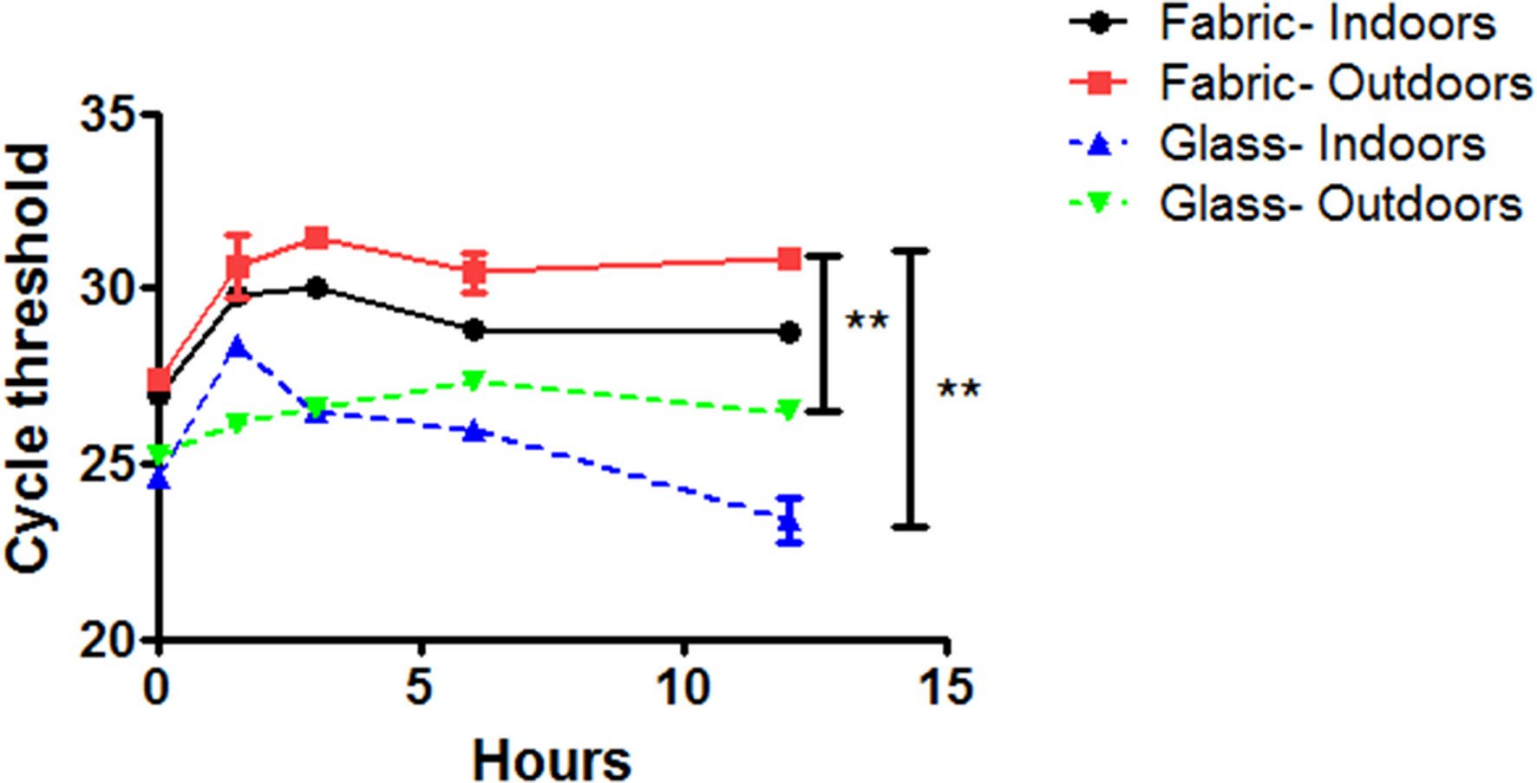
Cycle thresholds (CT) for detection of SARS-CoV-2 in the different contaminated materials in different conditions and time points. Analysis of variance with Bonferroni’s multiple comparison test. Statistical significance was set as p ≤ 0.05. **p ≤ 0.05. This result is representative of three independent experiments.

**Figure 2:**
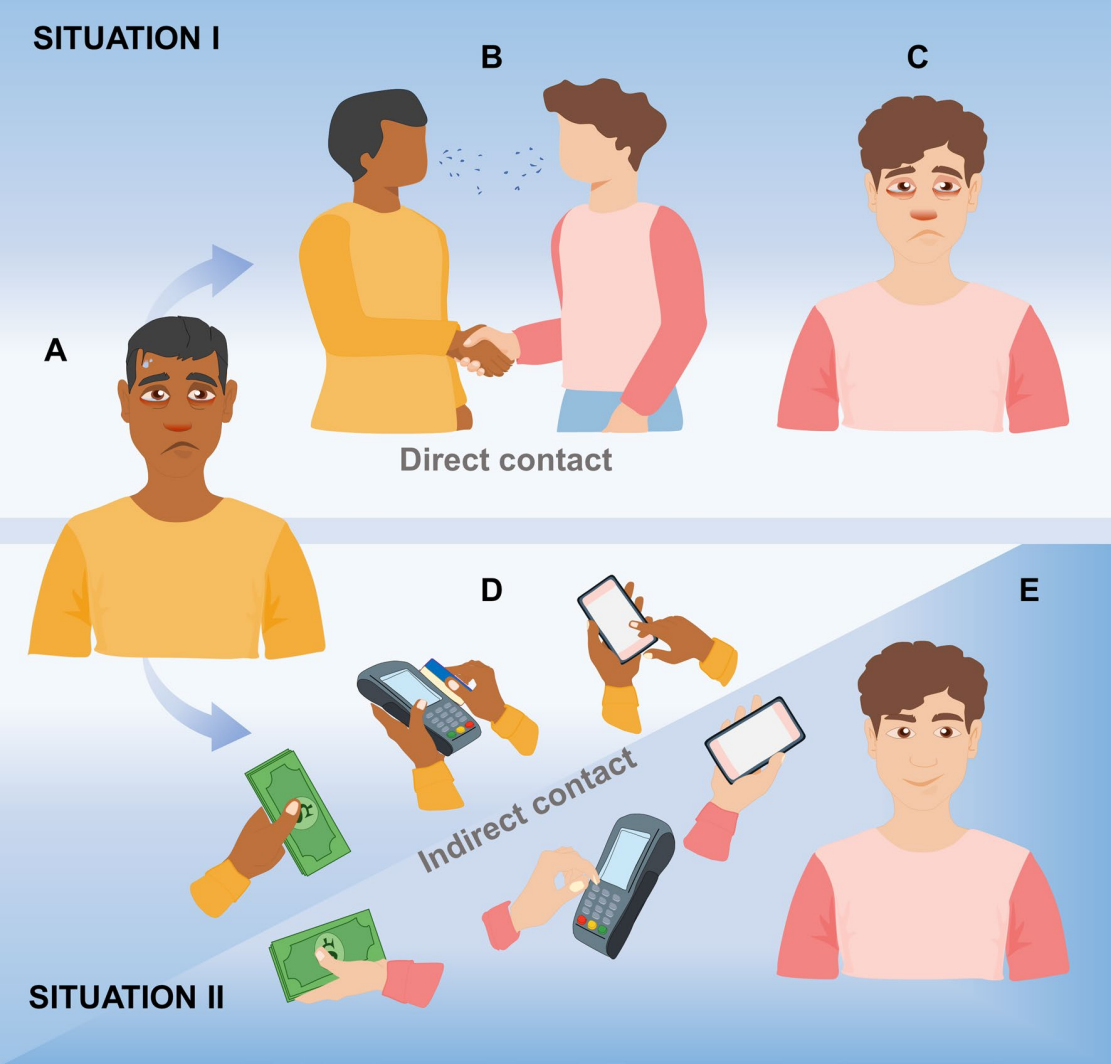
Direct contact as the main form of transmission of SARS-CoV-2. A, man with COVID-19. B, an infected man comes into direct contact with a healthy person and talks, neglecting the use of a mask and social distance. C, after contact with the infected person, the second man becomes infected with viruses. D, an infected man comes into contact with different inanimate objects. E, a healthy man comes into contact with inanimate objects that have been manipulated by an infected person and is not infected.

In order to rule out that the sampling method was not reliable to detect SARS-CoV-2 in environment and inanimate objects using RT-qPCR, we designed an experiment to verify sterile swabs are suitable to collect sources of nucleic acids. In addition, we aimed to verify if high temperatures and low humidity, which are typical of Barreiras city, were interfering in detection capacity. As shown in Fig. 2, SARS-CoV-2 RNA could be detected in both, glass and fabric, independently of conduction of the experiment in laboratory or outside conditions. Even with visible differences in temperatures and humidity in the two different conditions the detection of viral RNA by RT-qPCR was not prevented. The cycle threshold was significantly higher in fabric than in glass. This result indicates that the detection was significantly more sensible in glass (see Fig. 1). These results clearly show that sterile swabs collected sources of nucleic acids. Moreover, the sampling and detection of viral RNA were not affected by temperature or humidity. Collectively, these results indicate that our sampling method is reliable.

### Role of inanimate objects in transmission of SARS-CoV-2

Indirect transmission depends on the presence of a given infectious agent in inanimate objects and/or the environment. Our results show that SARS-CoV-2 was not present in sampled inanimate objects and environmental samples during the ascendant curve of COVID-19 cases in Barreiras city, Brazil. We also showed that our sampling method is reliable. As proposed in Fig. 2, together, these results indicate that inanimate objects and the environment did not contribute to COVID-19 transmission in Barreiras city during the period of study.

## Discussion

In this work we aimed to contribute to the understanding of the role of environment in the transmission of COVID-19. The literature contains reports showing detection of SARS-CoV-2 in hospital environments^13,14,22^. However, it has been recently reported that the risk of acquiring COVID-19 by touching contaminated surfaces in less than 5 in 10,000^20,23^. In addition, although SARS-CoV-2 RNA has been detected in hospital environment the virus could not be isolated in cell culture^22^. It is known that respiratory diseases caused by viruses such as Rhinovirus colds are not indirectly transmitted through fomites^24^. So, do we need to keep investing billions of dollars in disinfecting environments and inanimate objects in the COVID-19 pandemic?

Our results show that SARS-CoV-2 was not detectable in the environment or in fomites during its active circulation if human hosts in Barreiras city, Brazil. The detection of human RNAse P gene in samples indicates that human cells or their fragments were present in studied materials. We stress that we did not detect any trace of SARS-CoV-2 in samples studied here. We analyzed more than 400 samples collected in different occasions during the ascendant curve of COVID-19 cases in Barreiras city. These samples were collected from a market area with intense circulation of people and also from a reference health unit for COVID-19. In addition to the lack of SARS-CoV-2 in samples collected from cell phones, card machines, mask fronts and paper money from the market area, we did not detect viral RNA even from bedding and front masks of hospitalized COVID-19 patients. We must highlight that most of people were using masks during the samplings and that this can have limited the contamination of objects due to reduced virus levels exhaled into the air and depositing on surfaces. Although we have these striking results, one could argue that our sampling method was not recovering enough sources of nucleic acid for molecular detection by RT-qPCR.

To rule out that viral detection has been affected by the sampling method we designed an experiment to verify it. Although we have already shown that human RNAse P gene could be detected, the recovery of viral RNA in environmental or inanimate objects could be difficult. The amplification of RNAse P gene can be provided by using DNA as a template. However, amplification of SARS-CoV-2 targets can be provided only using RNA as a template in this situation. Our results indicate that the virus can be detected by RT-qPCR independently of time of exposure to environment until 12 h. In addition, detection is not prevented by variations in temperature or humidity. We could argue that the hostile clime of Barreiras with elevated temperatures and low percentages of humidity could be preventing molecular detection of SARS-CoV-2 in this study. Our results show that viral RNA could be detected even with a temperature of 43.8 °C and with exposure of 6 h at outdoors conditions the. We found only a difference in cycle thresholds for recovery from glass or fabric. The amplification of SARS-CoV-2 targets in samples recovered from fabric was significantly lower than those from glass. We hypothesize that adsorption of viral particles and/or viral RNA by fabric decreases the recovery of amplifiable RNA. Thus, detection of coronavirus RNA in bedding and front masks from COVID-19 patients can be limited or prevented by the characteristics of the fabric.

Here, we did not studied the same kind of inanimate objects previously reported as sources of SARS-CoV-2 RNA^13,14^. We investigated objects of high frequent contact with hands in the market area, such as card machines, paper money and cell phones. In the hospital environment we studied objects that we really expected to detect SARS-CoV-2 RNA, such as bedding and front masks of COVID-19 patients. But for our surprise, we did not detect any trace of viral RNA. However, as explained above, sampling from fabric can limit the detection of the viral RNA. Thus, our results are not necessarily representative of the hospital environment, although we have also studied cell phones and the air at the Eurico Dutra Hospital, which is the city reference health unit for COVID-19. It is important to highlight that our sampling was carried out in a mid-sized city, with less than 500,000 inhabitants and that detection of viral RNA can be limited by a low prevalence of COVID-19 cases if compared with those of great cities, such as Sao Paulo, Recife or New York. On the other hand, we used a highly sensitive and efficient method of molecular detection of SARS-CoV-2 RNA^25^. In addition, RT-qPCR assays used here had a limit of detection of 10 genome copies, regarding SARS-CoV-2. It corresponds to a CT value of 37. Moreover, we highlight that the sample size used in this study was adequate. Thus, it would be possible to detect viral RNA molecules if they were present in samples analyzed here.

Both the CDC (Center for Disease Control and Prevention) and the WHO (World Health Organization) state that the main form of infection by SARS-CoV-2 is through exposure to respiratory fluids from people carrying the virus. This can occur in three ways: (i) inhalation of respiratory droplets and aerosols; (ii) deposition of respiratory droplets in the nose, mouth or eyes; and finally, iii) touching the mucous membranes with hands contaminated by respiratory fluids that contain the virus^26–29^ The guidelines also state that respiratory fluids are released in the form of droplets during speech, coughing, singing, sneezing and exercising. Larger droplets are quickly deposited, while smaller ones and aerosols can remain in the air for hours. WHO states that any situation in which a person is in close proximity to another for long periods of time increases the risk of transmission. Indoor locations, especially settings where there is poor ventilation, are riskier than outdoor locations. Activities where more particles are expelled from the mouth, such as singing or breathing heavily during exercise, also increase the risk of transmission. According to WHO “Three C’s” are a useful way to think about this: crowed places, close-contact settings, especially where people have conversations very near each other and confined and enclosed spaces with poor ventilation. The risk of COVID-19 spreading is especially high in places where these “3Cs” overlap. CDC considers the risk of being infected through contact with contaminated surfaces or objects (fomites) as low.

In conclusion, our results indicate that, so far, fomites and the environment did not contribute to COVID-19 transmission in Barreiras city. Similar results can probably be found in other cities, mainly those with COVID-19 epidemiological scenarios similar to that of Barreiras city. Our study is a small piece indicating the possibility that fomites and the environment do not have an important role in COVID-19 transmission. We stress that physical distancing, wearing masks and other non-pharmacological interventions used to control other respiratory viral diseases are essential to control COVID-19 until we have the majority of populations vaccinated. Further studies are necessary to better understand the roles of fomites and the environment regarding COVID-19 transmission in the world scenario.

## Data Availability

Data will be provided under request.
